# Applicability of International Autoimmune Hepatitis Group (IAIHG) Scoring System for Autoimmune Hepatitis in Pediatrics

**DOI:** 10.3390/biology12030479

**Published:** 2023-03-21

**Authors:** Vorada Sakulsaengprapha, Paul Wasuwanich, Gayathri Naraparaju, Yelena Korotkaya, Supharerk Thawillarp, Kiyoko Oshima, Christine Karwowski, Ann O. Scheimann, Wikrom Karnsakul

**Affiliations:** 1 Division of Pediatric Gastroenterology, Hepatology, and Nutrition, Department of Pediatrics, Johns Hopkins University School of Medicine, Baltimore, MD 21205, USA; vorada@stanford.edu (V.S.); gayi3.gr8@gmail.com (G.N.); ckarwowski@caremount.com (Y.K.); ykorotkaya@gmail.com (C.K.); ascheima@jhmi.edu (A.O.S.); 2 College of Medicine, University of Florida, Gainesville, FL 32610, USA; p.wasuwanich@ufl.edu; 3 Department of Health Policy and Management, Bloomberg School of Public Health, Johns Hopkins University, Baltimore, MD 21205, USA; sthawil1@alumni.jh.edu; 4 Department of Pathology, Johns Hopkins University School of Medicine, Baltimore, MD 21205, USA; koshima3@jhmi.edu

**Keywords:** liver diseases, biopsy, pathology, public health

## Abstract

**Simple Summary:**

Autoimmune hepatitis (AIH) is a difficult liver disease to diagnose, and researchers developed the International Autoimmune Hepatitis Group (IAIHG) scoring system to aid the diagnosis of AIH. The scoring system was originally designed for adult patients; thus, we aim to evaluate the performance of this scoring system in children for accurately diagnosing AIH. We found liver biopsies were an essential component of the IAIHG scoring system and that specific liver biopsy features including interface hepatitis and predominant plasma cells were significantly associated with AIH. Incorporating liver biopsy data improves the performance of the IAIHG scoring system. Although, the degrees of importance of each specific biopsy feature are more distinct in the children population compared to those of the adult population. Overall, we determined that the IAIHG score is effective at diagnosing AIH in children, but could be improved.

**Abstract:**

Introduction: Many hepatologic pathologies mimic autoimmune hepatitis (AIH). Researchers developed the International Autoimmune Hepatitis Group (IAIHG) scoring system to compensate for the lack of specific diagnostic tests for AIH. The scoring system was not designed with pediatric patients in mind, so there are limits to its pediatric use. Additionally, there is limited information on the value of a liver biopsy in conjunction with its use. Methods: In this retrospective study, we evaluated the effect of liver biopsy scores on the IAIHG scoring system in patients that were 0–18 years old with suspected AIH. We also analyzed demographic data and laboratory values associated with a final AIH diagnosis. Results: We found that interface hepatitis and predominant plasma cells found during the biopsy were significantly associated with a final AIH diagnosis. We also found that abnormal laboratory values were associated with an AIH diagnosis. We found that IAIHG scores calculated post-liver biopsy showed a greater area under the receiver operating characteristic curve (AUROC) of 0.95, which was compared to 0.88 for the scores calculated before a liver biopsy. Including biopsy metrics lowered the optimized cutoff score and test specificity. Conclusion: Incorporating liver histopathological features improved the performance of the IAIHG scoring system. Further studies to identify other potential elements in liver histology may improve the performance metrics of the IAIHG test in the pediatric population.

## 1. Introduction

It is challenging for clinicians to diagnose autoimmune hepatitis (AIH) because there is no diagnostic gold standard for this disease. The International Autoimmune Hepatitis Group (IAIHG) developed a scoring system in 1992 and updated it in 1999. The scoring system uses clinical history, biochemistry, serologic tests for viral hepatitides, autoimmune markers, and liver histopathologic findings [1]. The 1999 update improved the ability to exclude a diagnosis of AIH in patients with autoimmune biliary diseases such as primary biliary cholangitis and primary sclerosing cholangitis [2,3]. Clinicians apply the IAIHG scoring system to patients with a high suspicion index for AIH. The results guide subspecialty referral decisions.

The overall diagnostic sensitivity and specificity for the IAIHG scoring system range from 97% to 100% and 44% to 87%, respectively [4].

However, multiple elements of the IAIHG scoring system lack relevance for pediatric patients. For example, alcohol intake is less relevant when one is evaluating children [5]. Further, gamma-glutamyl transpeptidase (GGT) is a more sensitive and specific indicator of cholestasis in pediatric biliary disorders than alkaline phosphatase (ALP) is. Growing children often have mild elevations in ALP levels due to bone-related longitudinal growth. Additionally, non-alcoholic fatty liver disease (NAFLD) is increasing worldwide across pediatric age groups. Overlapping clinical and biochemical presentations between NAFLD and AIH populations reduces the effectiveness of IAIHG scoring [6].

The study’s objective is to evaluate the relevance of liver histopathology features when they are used with IAIHG scoring to assess children with suspected AIH.

## 2. Material and Methods

### 2.1. Study Population

We conducted a retrospective study of patients from 0 to 18 years of age evaluated and treated at Johns Hopkins Children’s Center. These patients had an initial diagnosis of AIH, with or without sclerosing cholangitis or immune-mediated cholangitis, between 1 January 1990 and 24 May 2019. The study was approved by the Institutional Review Board at Johns Hopkins University School of Medicine. All patients were initially suspected of having autoimmune hepatitis with or without primary sclerosing cholangitis. Their final diagnosis as AIH (case group) was made based on clinical history, blood laboratory tests, liver histopathology, and their responses to AIH treatment. The control group consisted of patients under investigation for AIH who did not have a final diagnosis of AIH. We excluded patients with uncorrected coagulopathy at the time of diagnosis (and thus, with an increased bleeding risk) and patients who did not undergo a liver biopsy examination before treatment initiation. We also excluded patients with cholelithiasis and underlying immunodeficiencies such as agammaglobulinemia or severe combined immunodeficiency syndrome.

### 2.2. Data Collection

We collected demographics from the time of diagnosis (i.e., race, sex, and age). We also collected clinical data, including body mass index, fasting lipid panel, insulin level, hemoglobin A1C, serum ALP level, aspartate transaminase (AST) level, alanine transaminase (ALT) level, GGT level, serum total, and direct bilirubin levels, total immunoglobulin G (IgG) level, antinuclear antibody (ANA) level, anti-smooth muscle antibody (ASMA) level, anti-mitochondrial antibody (AMA) level, IgG subclasses, hepatitis viral markers, drug history, average alcohol intake, liver histology at diagnosis, other autoimmune diseases, family history of autoimmune diseases, other defined autoantibodies, and HLA-DR carrier status if they had been tested. Autoantibody tests were conducted at our institution using indirect immunofluorescence, and the results are reported in titers [7]. Positivity for an autoantibody was determined using cutoff values from our institution’s laboratory.

For each patient, we calculated a score using the IAIHG revised scoring system; the score was based on laboratory values, drug and alcohol use, histopathology, and response to therapy. Based on the score, patients were classified as definitely having AIH, probably having AIH, or other, both before and after the AIH treatment. Definitely having AIH requires scores of greater than 15 pre-treatment and 17 post-treatment, and probably having AIH requires scores of 10–15 pre-treatment and 12–17 post-treatment if they were receiving treatment for AIH [4]. Response to AIH treatment was determined using pre- and post-treatment IAIHG scores. In addition to the IAIHG scoring system, we also evaluated a more recently proposed, simplified AIH scoring system that has been externally validated in adult patients by Muratori et al. [8]. Histopathology data were reviewed by our senior pathologist, K.O. Histopathological features that were included in the IAIHG scoring system including interface hepatitis, predominant lymphoplasmacytic infiltrate, rosetting of liver cells, and biliary changes were of particular focus.

We based the diagnosis of sclerosing cholangitis on the findings of magnetic resonance cholangiopancreatography (MRCP) or endoscopic retrograde cholangiopancreatography (ERCP). We diagnosed overlap syndrome if the patient had clinical and histopathologic findings that were consistent with AIH and had sclerosing cholangitis confirmed by MRCP or ERCP.

We also collected IAIHG scores taken before and after liver biopsies to determine the impact of biopsies on the scores.

### 2.3. Statistical Analysis

We summarized data using frequencies with percentages or medians with interquartile ranges (IQRs). We analyzed associations using logistic regression. We constructed receiver operating characteristic (ROC) curves and the area under the ROC curve (AUROC). We used the ROC curves to determine optimal cutoffs for how well IAIHG scores predicted the final diagnosis of AIH. We defined optimal cutoffs as having the highest summation of specificity and sensitivity.

We tested normality with the Shapiro–Wilk test, where *p* < 0.05 indicated non-normal data. We reported non-normal data as the median and IQR. We performed analyses using Program R Version 3.2.3 (R Foundation for Statistical Computing, Vienna, Austria). We assumed that missing data were randomly missing. All reported *p*-values were two-sided, and *p* < 0.05 was considered to be statistically significant.

## 3. Results

We included a total of 61 patients in our study, including those in the control group (Table 1). The median age of this group was 13 (IQR: 8–16), with 32 (52%) of them being female. The race distribution was 51% White, 20% Black, and 30% other or unknown. From this cohort, we identified a total of 32 children who had a diagnosis of AIH (Table 1). Of the 32 children, 20 (63%) were female, and 12 (38%) were male. The median age was 14 years (IQR: 8–15), ranging from 1 to 17 years (Table 1). None of the patients were subjected to HLA-DR testing. There were 29 patients in the control group, and they had the following diagnoses: NAFLD (14; 48%), drug-induced liver injury (3; 10%), primary sclerosing cholangitis (2; 7%), parenteral nutrition-associated liver disease (2; 7%), and liver enzyme elevation with a normal liver biopsy (8; 28%).

**Figure 1:**
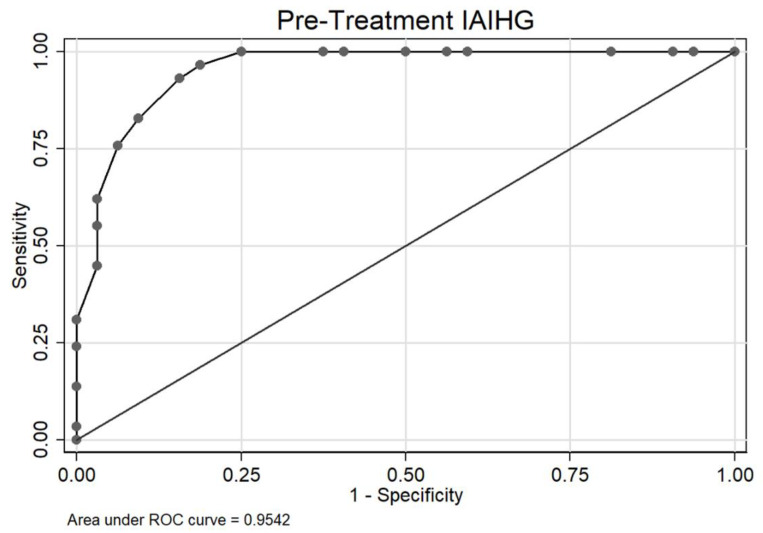
Receiver operating characteristic (ROC) curve for pre-treatment International Autoimmune Hepatitis Group (IAIHG) scores.

**Figure 2:**
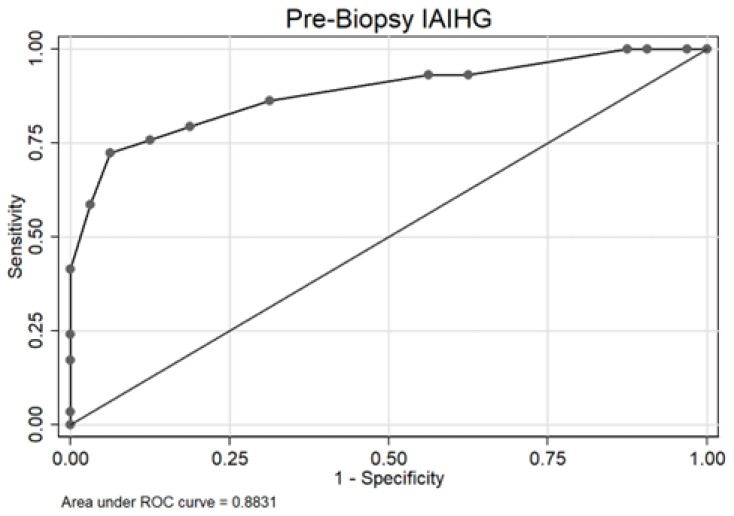
Receiver operating characteristic (ROC) curve for pre-biopsy International Autoimmune Hepatitis Group (IAIHG) scores.

We determined the AUROC for IAIHG score variants (Table 2, Figure 1 and Figure 2). The pre-liver biopsy IAIHG scores had an AUROC of 0.88 (95% CI: 0.80–0.97), and the pre-treatment scores (post-liver-biopsy) had an AUROC of 0.95 (95% CI: 0.91–1.00). We could not generate AUROC curves post-treatment, given the absence of AIH treatments in the control group. The optimized cutoff was ≥7 for the pre-treatment scores, with 97% sensitivity and 81% specificity. The optimized cutoff was ≥9 for the pre-liver-biopsy scores, with 72% sensitivity and 94% specificity. We also evaluated a simplified AIH scoring system and found the AUROC to be 0.91 (95% CI: 0.84–0.98). The optimized cutoff was ≥5, with 69% sensitivity and 97% specificity.

We analyzed the sensitivity and specificity of historic IAIHG cutoffs for both definite and probable AIH. Definite AIH cutoffs for the pre-treatment scores (>15) had 24% and 97% sensitivity and specificity, respectively. Probable AIH cutoffs for the pre-liver biopsy scores (10–15) had 59% and 97% sensitivity and specificity, respectively. Probable AIH cutoffs for the pre-treatment scores (10–15) had 52% and 94% sensitivity and specificity, respectively. We also evaluated the cutoffs provided by the creators of the simplified AIH scoring system, with a cutoff of ≥6 for probable AIH and a cutoff of ≥7 for definite AIH. Applying the probable cutoff yielded a sensitivity of 59% and a specificity of 97%, while the definite cutoff yielded a sensitivity of 31% and a specificity of 97%.

Certain race categories formed a risk factor for AIH (Table 3). More specifically, Black patients were more likely to be diagnosed with AIH than other groups were (OR: 7.89, *p* = 0.013), while patients who identified as Asian or an unknown race were not likely to have the diagnosis (OR: 0.07, *p* = 0.001). Age and sex were not associated with AIH diagnosis. None of the patients had a history of alcohol consumption.

Of note, two patients were younger than five years of age and were later diagnosed with very early onset inflammatory bowel disease. One of these patients was diagnosed with overlap syndrome after AIH treatment. One patient developed systemic lupus erythematosus after the AIH treatment.

Co-infection with hepatitis and other viruses was not associated with a final diagnosis of AIH (Table 3). Laboratory values such as ANA, ASMA, elevated total serum IgG, AST, GGT, ALP, total and direct bilirubin, hemoglobin, and INR were associated with a final diagnosis of AIH. In contrast, other values such as anti-LKM-1, AMA, p-ANCA, ALT, C-reactive protein, erythrocyte sedimentation rate, serum ceruloplasmin, alpha-1 antitrypsin, white blood cell count, and platelet count were not found to be significantly associated with an AIH diagnosis (Table 3).

Of those with a final diagnosis of AIH, the histopathologic report indicated that 13 patients (41%) had interface hepatitis, 23 (72%) had predominant plasma cells, 8 (25%) had biliary changes, and 15 (47%) had hepatic fibrosis and regenerating nodules. Of these, interface hepatitis and predominant plasma cells were associated with a final diagnosis of AIH (Table 3).

The feature with the highest sensitivity and specificity was predominant plasma cells (72% and 97%, respectively) (Table 4). Biopsies interpreted as being consistent with AIH had the same sensitivity and specificity (Table 4).

## 4. Discussion

Autoimmune hepatitis (AIH) is a chronic, progressive immune-mediated inflammatory liver disorder. Its initial presentation can be acute, subacute, or chronic. AIH is considered to be rare in children and adolescents, so it can be missed due to a low index of suspicion [9]. There are three main types of AIH that can be distinguished by liver autoantibodies [10]. Type 1 (AIH-1) is characterized by antinuclear (ANA) or anti-smooth muscle (ASMA) antibodies. Type 2 (AIH-2) is characterized by antibodies to liver-kidney microsome type 1 (anti-LKM-1) or antibodies to liver cytosol type 1 (anti-LC1). Type 3 (AIH-3) is characterized by anti-soluble liver antigen/liver-pancreas antibodies. AIH is extremely rare in patients under two years of age. The highest incidence occurs between 10 and 30 years old, affecting females more than it does males [11,12,13]. While the AIH can occur in all decades of life, there are several notable distinctions in the clinical and laboratory features. AIH in elderly patients tends to be more asymptomatic, more likely to be associated with a positive ANA, and more likely to be associated with HLA-DR4 [14]. It has been theorized that the high frequency of ANA in elderly/older patients reflects the increased incidence of autoantibodies with age in the normal population [14].

AIH is clinically characterized by hypergammaglobulinemia, elevated liver enzymes, the presence of autoantibodies, and histological changes. Its diagnosis is confirmed by clinical findings, laboratory and histopathology tests, and the exclusion of other causes of chronic liver disease [11,12]. In our study, high laboratory values (i.e., serum IgG, ANA, and ASMA) were associated with a final diagnosis of AIH. ANA, ASMA, and anti-LKM-1 have been found to constitute the conventional serological repertoire for an AIH diagnosis [15]. ANA has been found to be present in 80% of White North American adults with AIH at presentation; 63% have positive ASMA and 3% have positive anti-LKM-1 [15], which may reflect our findings of ANA and ASMA being significantly associated with an AIH diagnosis. In our study, only one (3%) patient from the AIH group had anti-LKM-1, which is a similar to rate to that reported in the adult AIH population; the low case number of patients with anti-LKM-1 is the primarily reason for lack of statistical significance in that variable in our study. The low prevalence of anti-LKM-1 in our study could be potentially explained by distinct genetic backgrounds in different geographic locations. Muratori et al. reported that anti-LKM-1 rarely occurs in North America, which is likely related to the lower frequency of HLA DR7 in North America compared to that in the Italian population [16].

Autoantibodies may be negative or present at low titers at the disease onset stage [17]. Yet, autoantibodies may become detectable at a later follow-up with acute or fulminant presentations before liver biopsy procedures. Measuring autoantibody titers during this later period may improve both the sensitivity and specificity.

Laboratory values such as AST, GGT, ALP, total and direct bilirubin, hemoglobin, and INR were significantly associated with a final diagnosis of AIH (Table 3). Occasionally, AIH can sometimes present with a cholestatic picture [18]. Additionally, certain laboratory values, such as ALP and GGT, may also be indicative of overlapping features of AIH with other entities such as primary biliary cholangitis. This mixed picture emphasizes the importance of including diagnostic parameters such as history, biochemical markers, and biopsy findings reflected in scores.

In our study, we investigated both ALT and AST; however, only AST was found to be significantly associated with AIH. While ALT and AST are related enzymes, their distribution across the body is unique. AST isoenzymes are present in the mitochondria and cytosol of cells and can be found in the liver, skeletal muscle, cardiac muscle, kidneys, brain, pancreas, lungs, leukocytes, and red blood cells. On the other hand, ALT is a cytosolic enzyme that mainly occurs in significant concentrations in the liver. As such, ALT has been generally considered to be more sensitive and specific for liver disease and injury [19]. Despite that, AST is more commonly used in AIH diagnosis and disease monitoring [20]. A major reason is the shorter half-life of AST (approximately 17 h) compared to that of ALT (approximately 47 h) [21]. Because AIH is an ongoing and progressive liver injury, the shorter half-life of AST makes it relatively more useful than ALT is. AST is superior for measuring the current state of liver inflammation and injury, being more associated with diseases where liver inflammation and injury are continuous and not intermittent. As such, even though both ALT and AST levels were elevated in our cohort, AST elevations were more likely to be specific to pediatric patients with AIH rather than the controls with non-AIH liver disease.

Diseases such as hepatitis A, B, C, E, Wilson’s Disease, NAFLD, and drug-induced liver injury (DILI) share histopathologic features with AIH, including false-positive liver autoantibodies [17]. Excluding these diseases is vital before a diagnosis of AIH can be made. Some of these pathologies were present in a minority of our AIH cohort, but were not the primary etiology of liver disease. In order to improve the exclusion ability of the IAIHG scoring system, we propose the utilization of RUCAM (Roussel Uclaf Causality Assessment Method), a scoring system commonly used to quantify the likelihood of DILI [22]. Currently, the IAIHG scoring system only includes a binary option of hepatotoxic drug history; the integration of RUCAM could potentially improve the performance of the IAIHG scoring system by quantifying the likelihood of significant drug involvement in the liver disease or could be used to exclude or confirm hepatotoxic or potentially hepatotoxic drugs for liver disease involvement.

NAFLD, a common liver disease, is associated with elevated ASMA and total serum IgG levels [23]. In NAFLD, we see a female predominance, elevated autoantibodies, the presence of ANA and ASMA, polyclonal hypergammaglobulinemia, interface hepatitis on biopsy, and a good response to immunosuppression [15,24,25]. NAFLD patients could be falsely diagnosed with AIH due to these overlapping characteristics.

Histopathologic findings from a liver biopsy are standard criteria for diagnosing AIH in children [17,18]. We used a pre-liver-biopsy score to determine whether the IAIHG scoring system was sensitive enough to predict AIH diagnosis without information from a liver biopsy. We achieved an AUROC of 0.88, with a sensitivity of 72% and specificity of 94%, using an optimized cutoff score of ≥9 (Table 2).

Pre-treatment IAIHG scores using histopathologic features outperformed the pre-liver-biopsy scores, with an AUROC of 0.95, sensitivity of 97%, and specificity of 81%, using a lower optimized cutoff score of seven (Table 2). This provides strong evidence that using liver biopsy within the IAIHG system helps to predict AIH diagnosis in children with abnormal liver enzymes. When one is including biopsy findings, the scoring system performed well for ruling in AIH, and it was even better at ruling out non-AIH diagnoses. As the IAIHG scoring system is rather extensive, a simplified scoring system for AIH has been proposed. This scoring system was developed by Hennes et al. in 2008 [26], and it was externally validated by Muratori et al. the following year in an Italian adult population [8]. Muratori et al. reported the overall sensitivity and specificity for the AIH diagnosis at a cutoff score of ≥6 to be 91.8% and 94.3%, respectively [8]. In our pediatric cohort, while we reported similar specificities at the cutoff of ≥6, the sensitivity was found to be notably lower in our cohort, 59%. However, with an AUROC of 0.91 in our study, this simplified AIH score has potential for application in the pediatric population. With an optimized cutoff of ≥5, the sensitivity was increased to 69%, while maintaining a specificity of 97%. A lower cutoff for this simplified AIH scoring should be considered when one is applying it to the pediatric population. Nevertheless, as the sensitivity is relatively low even when it has been optimized, the score may be more useful for confirming a diagnosis of AIH rather than ruling out non-AIH diagnoses.

We found that interface hepatitis and a predominance of plasma cells predicted an AIH diagnosis (Table 3), which is consistent with previous studies identifying these features as hallmarks of AIH [15]. Interface hepatitis is characterized by dense inflammatory infiltrates composed of lymphocytes and plasma cells. However, it is important to note that histopathological features are not diagnostic in isolation; while they are common in pediatric patients with AIH, they are not exclusive to AIH [15,17,27]. Compared to the current IAIHG scoring system, which provides more weight to interface hepatitis than predominantly lymphoplasmacytic infiltration, our study found the opposite, with a predominance of plasma cells being more strongly associated with AIH.

Emperipolesis, the presence of an intact cell within another cell, is another histopathological feature that has been widely described in patients with AIH, although it is not explicitly included in the IAIHG scoring system. Miao et al. conducted a retrospective histological evaluation of 101 patients with AIH with 184 controls using confocal staining for CD4, CD8, CD56, CK8/18, and cleaved caspase-3 [27]. They reported emperipolesis in 65% of the patients with AIH using hematoxylin and eosin-stained slides, which was significantly higher than in the patients with a drug-induced liver injury (26%), primary biliary cirrhosis (18%), and chronic hepatitis B (15%). Additionally, they found that emperipolesis was associated with more advanced fibrosis and more severe necroinflammatory features. The emperipolesis of CD8 T cells induced cleaved caspase-3 expression and was prominent in areas of apoptosis. Emperipolesis is a characteristic feature of AIH, which is often seen in conjunction with interface hepatitis, plasmocytic infiltration, and hepatocyte rosetting and is associated with more severe necroinflammatory and fibrotic changes. Emperipolesis is predominantly mediated by CD8 T cells in AIH, and it appears to induce apoptosis and may be another mechanism of autoimmune-mediated hepatocyte injury. Miao et al. reported that the combination of emperipolesis with interface hepatitis, plasma cell infiltrates, and hepatocyte rosettes achieved a sensitivity of 81% and a specificity of 84% for diagnosing AIH [27]. However, a detailed look at the data published from many studies suggested that these two features carry a lower sensitivity due to difficulties in being identified or determined by light microscopy. Further study using immunostaining of CD8 T cells, along with confocal or electron microscopy, may help to assess the importance of emperipolesis.

In recent years, novel non-invasive biomarkers for AIH have been reported, but none of them have yet become part of routine clinical practice nor have replaced the liver biopsy. These biomarkers include adenosine deaminase, cytokeratin-18 death marker m65, transforming growth factor-ß1, tumor necrosis actor family B-cell activating factor (BAFF), anti-asialoglycoprotein receptor, FOXP3/RORɣt ratio, DNAse 1, ferritin, CD74:MIF ratio, and the vitamin D receptor [28].

The study was limited by a small sample size, variability in laboratory data, and its retrospective nature. By increasing sample size, the true effect of other factors such as race may be further elucidated as well; race is increasingly being recognized as a social construct, and the differences seen in this study may be more representative of socioeconomic status rather than a true biological difference.

Given the lack of HLA data, we could not determine their significance in the IAIHG scoring system. Currently, HLA data are optional additional parameters of the score calculator. To improve the efficacy and efficiency of the IAIHG scoring system, follow-up research should gather additional data regarding the importance of including or excluding the HLA status.

## 5. Conclusions

This study provides evidence of the utility of the IAIHG scoring system in the pediatric population and the importance of liver histopathology from biopsy for confirming the diagnosis of AIH and excluding other diagnoses, but not all liver histopathological features were equally predictive of AIH, and weights may need to be adjusted for the pediatric population. While the IAIHG scoring system includes some parameters that are not applicable to the pediatric population, such as alcohol use, many of its parameters were significantly associated with an AIH diagnosis.

Further studies are needed to identify other elements related to liver histopathology. Studies on using HLA data to modify the IAIHG score could also increase the score’s specificity for diagnosing AIH in pediatric patients.

## Figures and Tables

**Table 1:** Patient demographics.

Characteristics	All Cases	AIH Cases	Control Cases
Total Cases, N	61	32	29
Age, Years, Median (IQR)	13 (8–16)	14 (8–15)	12 (10–16)
Sex			
Male, N (%)	29 (48)	12 (38)	17 (59)
Female, N (%)	32 (52)	20 (63)	12 (41)
Race			
White, N (%)	31 (51)	19 (59)	12 (41)
Black, N (%)	12 (20)	11 (34)	1 (3)
Other/Unknown, N (%)	18 (30)	2 (6)	16 (55)

**Table 2:** Receiver operating characteristic (ROC) curve analysis and optimized cutoffs with sensitivity and specificity.

Biomarker	AUROC (95% CI)	Standard Error	Optimized Cut-Off	Sensitivity	Specificity
Pre-liver biopsy IAIHG score	0.88 (0.80–0.97)	0.04	≥9	72%	94%
Pre treatment IAIHG score with HLA	0.95 (0.91–1.00)	0.02	≥7	97%	81%
Post treatment IAIHG score with HLA	N/A	N/A	N/A	N/A	N/A
Simplified AIH score	0.91 (0.84–0.98)	0.04	≥5	69%	97%

AUROC = area under the receiver operating characteristic curve; IAIHG = International Autoimmune Hepatitis Group; HLA = human leukocyte antigen.

**Table 3:** Odds ratios of risk factors for autoimmune hepatitis (AIH) in all study patients.

Risk Factors		Odds Ratio (95% CI)	*p*-Value
*Demographics*			
Age (Year)	≥12 (vs. <12)	0.74 (0.26–2.06)	0.562
Sex	Female (vs. Male)	2.10 (0.76–6.00)	0.155
Race	White (vs. Other)	1.82 (0.66–5.13)	0.248
	Black (vs. Other)	7.89 (1.83–55.15)	0.013
	Asian/Unknown (vs. Other)	0.07 (0.01–0.30)	0.001
*Biopsy*			
Consistent with AIH	Yes/No	66.41 (11.44–100.00)	<0.001
Biliary Changes	Yes/No	1.72 (0.48–6.52)	0.407
Interface Hepatitis	Yes/No	21.88 (3.82–100.00)	0.004
Predominant Plasma Cells	Yes/No	39.38 (9.13–100.00)	<0.001
Fibrosis	Yes/No	0.72 (0.26–1.96)	0.518
*Clinical*			
Autoimmune Disease in Family	Yes/No	1.09 (0.39–3.00)	0.873
Recent Hepatotoxic Drug Use	Yes/No	3.50 (0.68–26.19)	0.158
6-Mercaptopurine Therapy	Yes/No	8.31 (2.49–33.76)	0.001
Steroid Therapy	Yes/No	100.00 (24.10–100.00)	<0.001
Ulcerative Colitis	Yes/No	1.67 (0.26–13.46)	0.589
*Viral Hepatitis Screen*			
Hepatitis A Virus	Yes/No	3.43 (0.40–72.25)	0.302
Hepatitis B Virus	Yes/No	N/A	N/A
Hepatitis C Virus	Yes/No	N/A	N/A
Hepatitis E Virus	Yes/No	N/A	0.996
Herpes Simplex Virus I/II	Yes/No	1.33 (0.12–31.69)	0.825
*Laboratory*			
ANA	Yes/No	5.70 (1.88–19.02)	0.003
ASMA	Yes/No	3.58 (1.17–12.09)	0.031
Anti-LKM-1	Yes/No	N/A	0.991
AMA	Yes/No	0.81 (0.03–21.25)	0.882
p-ANCA	Yes/No	0.78 (0.08–7.86)	0.822
IgG (mg/dL)	>1600 (vs. ≤1600)	16.00 (2.65–100.00)	0.012
AST (U/L)	>40 (vs. ≤40)	4.13 (1.10–20.20)	0.049
ALT (U/L)	>56 (vs. ≤56)	1.96 (0.59–7.24)	0.285
GGT (U/L)	>30 (vs. ≤30)	4.41 (1.30–17.95)	0.024
Alkaline Phosphatase (U/L)	>140 (vs. ≤140)	5.47 (1.50–26.53)	0.017
Total Bilirubin (mg/dL)	>1.2 (vs. ≤1.2)	9.21 (2.75–37.67)	<0.001
Direct Bilirubin (mg/dL)	>0.3 (vs. ≤0.3)	7.34 (2.11–30.85)	0.003
Creatine Kinase (U/L)	>200 (vs. ≤200)	2.34 (0.35–19.32)	0.379
C- Reactive Protein (mg/dL)	>0.8 (vs. ≤0.8)	1.02 (0.27–3.87)	0.975
Hemoglobin (g/dL)	<12.0 (vs. ≥12.0)	4.18 (1.47–12.68)	0.009
INR	>1.1 (vs. ≤1.1)	4.06 (1.27–14.74)	0.023
Erythrocyte Sedimentation Rate (mm/h)	>20 (vs. ≤20)	3.95 (0.97–20.47)	0.070
White Blood Count (/cu mm)	>11,000 (vs. ≤11,000)	0.57 (0.14–2.14)	0.416
Platelet Count (K/cu mm)	<150 (vs. ≥150)	8.09 (1.00–100.00)	0.061
BMI (kg/m^2^)	>24.9 (vs. ≤24.9)	0.22 (0.07–0.65)	0.009

ALT = alanine transaminase; AMA = antimitochondrial antibodies; ANA = antinuclear antibodies; Anti-LKM-1 = Anti-Liver/Kidney Microsomal Type 1; ASMA = Anti-Smooth Muscle Antibody; AST = aspartate transaminase; BMI = body mass index; GGT = Gamma-Glutamyl Transferase; IgG = Immunoglobulin G; INR = international normalized ratio; p-ANCA = Perinuclear Anti-Neutrophil Cytoplasmic Antibodies. N/A indicates insufficient data.

**Table 4:** Sensitivity and specificity values for autoimmune hepatitis (AIH) biopsy parameters.

Biopsy Data	Sensitivity	Specificity
Consistent with AIH	72%	97%
Biliary changes	24%	84%
Interface hepatitis	41%	97%
Predominant plasma cells	72%	94%
Fibrosis	45%	47%

## Data Availability

The data presented in this study are available on request from the corresponding author. The data are not publicly available due to patient confidentiality.
