# Correction: Sakulsaengprapha et al. Applicability of International Autoimmune Hepatitis Group (IAIHG) Scoring System for Autoimmune Hepatitis in Pediatrics. *Biology* 2023, *12*, 479

**DOI:** 10.3390/biology12050727

**Published:** 2023-05-16

**Authors:** Vorada Sakulsaengprapha, Paul Wasuwanich, Gayathri Naraparaju, Yelena Korotkaya, Supharerk Thawillarp, Kiyoko Oshima, Christine Karwowski, Ann O. Scheimann, Wikrom Karnsakul

**Affiliations:** 1 Division of Pediatric Gastroenterology, Hepatology, and Nutrition, Department of Pediatrics, Johns Hopkins University School of Medicine, Baltimore, MD 21205, USA; vorada@stanford.edu (V.S.); gayi3.gr8@gmail.com (G.N.); ykorotkaya@gmail.com (Y.K.); ckarwowski@caremount.com (C.K.); ascheima@jhmi.edu (A.O.S.); 2 College of Medicine, University of Florida, Gainesville, FL 32610, USA; p.wasuwanich@ufl.edu; 3 Department of Health Policy and Management, Bloomberg School of Public Health, Johns Hopkins University, Baltimore, MD 21205, USA; sthawil1@alumni.jh.edu; 4 Department of Pathology, Johns Hopkins University School of Medicine, Baltimore, MD 21205, USA; koshima3@jhmi.edu

## Text Correction

There was a spelling mistake in the original publication [1]. A correction has been made to ***Title***.

“Applicability of International Autoimmune Hepatitis Group (IAIHG) Scoring System for Autoimmune Hepatitis in Pediatrics”

## Text Correction

There was a spelling mistake in the original publication [1]. A correction has been made to ***Simple Summary***.

“We found liver biopsies were an essential component of the IAIHG scoring system and that specific liver biopsy features including interface hepatitis and predominant plasma cells were significantly associated with AIH.”

## Text Correction

There was a missing part in the original publication [1]. A correction has been made to ***Discussion***, the newly added sentence appears below.

“However, a detailed look at the data published from many studies suggested that these two features carry a lower sensitivity due to difficulties in being identified or determined by light microscopy. Further study using immunostaining of CD8 T cells, along with confocal or electron microscopy, may help to assess the importance of emperipolesis.”

The authors state that the scientific conclusions are unaffected. This correction was approved by the Academic Editor. The original publication has also been updated.
